# Nepalese Cancer Patients’ Willingness to Pay for Improved Quality of Life: A Choice Experiment Study

**DOI:** 10.3390/healthcare13141645

**Published:** 2025-07-08

**Authors:** Adnan Shahid, Alok Bohara

**Affiliations:** 1 Department of Social and Behavioral Sciences, Richard Bland College of William & Mary, Petersburg, VA 23805, USA; 2 Department of Economics, University of New Mexico, Albuquerque, NM 87106, USA; bohara@unm.edu

**Keywords:** quality of life, cancer, willingness to pay, discrete choice experiment

## Abstract

**Background/Objectives**: In Nepal, cancer, among non-communicable diseases, has a high mortality rate. The disease significantly affects patients’ quality of life (QoL). This study aims to identify key attributes of QoL and explore patients’ preferences regarding these attributes. **Methods**: We implement a discrete choice experiment (DCE) survey to understand cancer patients’ preferences for different attributes of QoL, their willingness to pay for improved QoL, and their preference heterogeneity. This study innovatively uses the EuroQol measure in a DCE setting to elicit the patients’ preferences and their willingness to pay. **Results**: Using a random parameter logit model, we find that cancer patients prefer lower levels of pain and higher levels of performing usual activities. Overall, we find that cancer patients are willing to pay a total amount of about NRS 2.6 million [about USD 26,000] for improved quality of life. Our analysis also shows that preference heterogeneity exists among cancer patients, and the presence of uncertainty in the preferences of patients does not affect the results. **Conclusions**: This study sheds light on the preferences and willingness to pay for improved quality of life among cancer patients in Nepal. Understanding these preferences can inform healthcare policy and resource allocation decisions aimed at improving the QoL of cancer patients in the region.

## 1. Introduction

Cancer is a leading global cause of mortality, with approximately 19.3 million new cases and 10 million deaths recorded in 2020 [1,2,3]. Disturbingly, the International Agency for Research on Cancer (IARC) predicts a 29 percent increase in new cancer cases and a 46 percent rise in deaths as a percentage of the total population by 2040. While the disease is more prevalent in developed economies, developing economies are closing the gap very rapidly [4]. Despite lower prevalence, these regions have the highest death rates, with about 70 percent of global cancer deaths [2]. This stark contrast results from an aging population, a key factor in global cancer trends, and the vital role of early detection and prevention. Developed economies have made strides in preventing some cancer types, with declines in incidence and mortality due to early detection and access to advanced therapies. Conversely, increased cancer mortality in developing economies results from a lack of prevention, late detection, or inadequate treatment facilities, with roughly 80 percent of patients facing incurable cancer at diagnosis [5].

Nepal, a low-income country of 27 million people, has seen a shift from a high prevalence of communicable to non-communicable diseases (NCDs). Presently, it grapples with a higher age-standardized death rate from NCDs, with cancer ranking among the top four. The 2018 WHO Nepal statistics show that 14,300 individuals died of cancer, with more deaths among females. Lacking a population-based cancer registry, the country relies on hospital-based data, estimating 8000 to 10,000 new cases annually, which is expected to increase in the future [6,7,8]. Following global patterns, common cancers include lung cancer in males and cancer of the cervix/uterus in females [9]. Contributing factors encompass tobacco use, alcohol consumption, dietary changes, and rapid urbanization [10].

In Nepal, the economic burden of cancer is tremendous, involving high out-of-pocket treatment costs that create barriers to healthcare access [11]. Natural disasters in the country, mainly earthquakes, destroy infrastructure and further limit access to healthcare facilities [12,13,14]. The disease can cause hospitalization, reducing mobility, long-term disability, and death, which further deteriorates patients’ QoL [15]. Besides physical suffering, many other factors affect patients’ QoL, including the knowledge of having a life-threatening disease, causing anxiety and mental depression, and aging [16,17]. Despite these challenges, a holistic approach focusing on social, psychological, emotional, and mental dimensions can enhance overall quality of life for cancer patients [17,18].

The primary objective of this study is to understand how Nepalese cancer patients value improvements in their quality of life and to quantify the economic trade-offs they are willing to make to achieve better well-being. To this end, we employ a discrete choice experiment to capture patients’ preferences and estimate their willingness to pay for enhanced well-being. Following EuroQol, we evaluate patients’ QoL using five factors: pain, depression, mobility, self-care, and usual activities. We believe that patients derive utility from each of these factors, which change as factor levels vary. Using these QoL attributes in our experimental setup, we assess patients’ QoL and their willingness to pay for improved quality of life. The improved QoL includes the best level of each attribute. Patient’s preference and willingness to pay for QoL attributes may vary based on cancer type and disease stage; thus, we also estimate marginal willingness to pay (MWTP) for each attribute level.

## 2. Materials and Methods

### 2.1. Context and Data Collection

This study uses primary data collected through a field survey conducted from May to July 2018 in four hospitals in Nepal, with a focus on three major cancer treatment hospitals that receive the highest patients annually. We administered the survey in two dedicated cancer hospitals and two general hospitals. The survey targeted both cancer and non-cancer patients, and participants were randomly selected from inpatient and outpatient departments.

While this paper primarily focuses on cancer patients, for comparison, we also consider non-cancer patients. The cancer cohort spans various types, from lung to cervix/uterus and more, while the non-cancer group includes those grappling with similarly severe conditions like Diabetes and Chronic Obstructive Pulmonary Disease (COPD). We administered the survey to both inpatient and outpatient individuals aged 18 and above, encompassing a range of aspects, including quality of life. Beyond the discrete choice experiment, we asked patients about their domestic and social life, as well as their demographics. We rigorously pretested the questionnaire at Bhaktapur Cancer Hospital and incorporated patients’ feedback that shaped the final version. The survey was designed in English but was subsequently translated and administered in Nepali. An interviewer administered the questionnaire and ensured that each participant had provided consent before proceeding. To obtain a representative sample, we aimed to survey as many patients as possible within our budget and time constraints. Ultimately, we collected the individual-level data from 1310 respondents, comprising 70% cancer patients and 30% non-cancer patients; all responded to the three choice tasks, resulting in 3930 observations for analysis.

### 2.2. Discrete Choice Experiment Design

This study uses DCE methodology to estimate patients’ willingness to pay for various attributes of quality of life. DCE relies on the idea that individuals derive utility not from the good presented but from its underlying attributes [19]. In this context, individuals are presented with different alternatives, each featuring varying levels of the pre-selected attributes. Respondents then choose their preferred alternative, assuming each choice provides higher utility than the rejected option. This allows us to model the probability of the chosen alternative in terms of the attribute levels [20].

**Figure 1:**
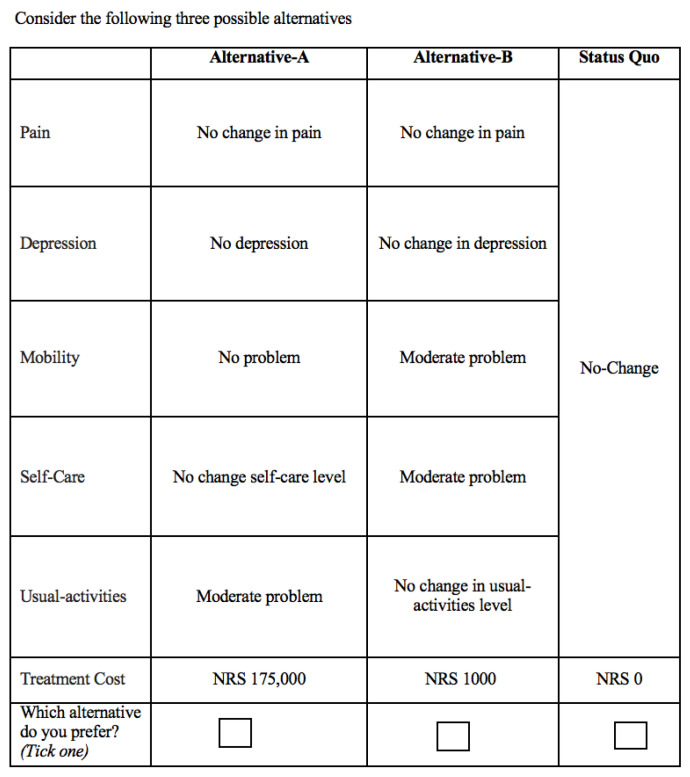
Sample choice set.

We presented three alternatives in a choice set to each respondent, with two differing based on attribute levels and the third representing the status quo. In our survey, we used five dimensions of the EuroQol, EQ-5D, a pre-tested and well-established instrument for assessing quality of life. These dimensions include pain, depression, mobility, self-care, and usual activities; however, we adjusted their levels. We used only two of the desired levels of each attribute while making changes to the definition and interpretation of the third level. Additionally, we used cost as an essential attribute, representing the fee individuals would pay for the selected alternative. While the first five attributes have three levels each, cost has thirteen levels determined following debriefings with oncologists, patients, and other coordinating doctors in the field to ensure the values reflected realistic and context-appropriate ranges. The attributes and their respective levels are presented in Table 1. A sample choice set is shown in Figure 1.

### 2.3. Empirical Strategy

Our study uses the random utility model (RUM) to understand how patients make choices. This model assumes that every patient gains a certain level of satisfaction from each option presented, but this satisfaction is not directly visible. We can, however, estimate most of this satisfaction by looking at the features of the option and the patient’s socio-economic characteristics. At the same time, we recognize that some part of a patient’s decision is unpredictable. The model assumes that people choose the option that gives them the most benefit. Since part of the decision-making process is random, we estimate the likelihood that a person will prefer one option over another [21,22].

### 2.4. Econometric Model

Drawing on the previous literature, our basic random utility model can be shown as

(1) $$\begin{array}{l} U_{njt}=\beta_{0}+\beta_{1}P_{M_{njt}}+\beta_{2}P_{N_{njt}}+{\beta_{3}D_{M_{njt}}}_{3}+\beta_{4}D_{N_{njt}}+\beta_{5}M_{M_{njt}}\\ \;\;\;\;\;\;\;\;\;\;\;\;+\beta_{6}M_{N_{njt}}+\beta_{7}SC_{M_{njt}}+\beta_{8}SC_{N_{njt}}+\beta_{9}UA_{M_{njt}}+\beta_{10}UA_{N_{njt}}+\beta_{11}Cost_{njt}\\ \;\;\;\;\;\;\;\;\;\;\;\;+\varepsilon_{njt} \end{array}$$

where P, D, M, SC, and UA represent pain, depression, mobility, self-care, and usual activities, respectively; the subscripts M, N, n, j, and t refer to moderate problem, no problem, individual, choice set alternative, and choice set, respectively; for example, $P_{M}$ and $D_{N}$ refer to moderate pain and no depression. The coefficients $\beta_{1}$ to $\beta_{10}$ represent the marginal utilities of different attribute levels, and $\beta_{0}$, the intercept or alternative-specific constant (ASC), represents the utility of the status quo, unique to each individual’s current quality of life.

Equation (1) above is first estimated using the conditional logit model (CL). However, one of the underlying assumptions of the model, Independence from Irrelevant Alternative (IIA), is not satisfied [23]; therefore, we employ the random parameters (or mixed) logit model (RPL). This allows for random taste variation, unrestricted substitution patterns, and correlation in unobserved factors over time [22]. After estimating Equation (1) using the mixed logit model, we use the t-statistic for selecting the random parameters [24].

### 2.5. Welfare Measure

Our goal is to calculate the marginal willingness to pay (MWTP) of cancer and non-cancer patients for different quality of life dimensions. The MWTP is the amount of income that compensates for a marginal increase in the quality of the non-market good. This helps us understand the relative importance patients place on different aspects of care. We can calculate the MWTP by comparing how strongly patients respond to improvements in quality of life against how they respond to changes in cost. Mathematically, $MWTP=-\frac{\beta_{attribute}}{\beta_{cost}}$ [20,25]. We can also calculate the combined willingness to pay for a number of attributes by summing all the attribute coefficients and dividing by the cost coefficient [26].

## 3. Results

### 3.1. Summary Statistics

Table 2 summarizes the socio-demographics of the patients. Seventy percent are cancer patients, mostly outpatients (84%). The majority of our sample consists of females (52%) and married (80%) individuals with an average age of 52 years. Mostly, the respondents are Brahmin, Chhetri, or Janajati, with incomes between NRS 10,000 and NRS 30,000. Among cancer patients, lung, breast, stomach, esophageal, head and neck, brain, and cervical cancers are prevalent. Breast and cervical cancers account for 36%, with breast (31.0%) and cervical (28.0%) being most common in females. Males primarily suffer from lung (17.0%), head, neck, and brain (15.0%), and oral and nasopharynx (10.0%) cancers.

### 3.2. Main Results

Table 3 and Table 4 present the utility and marginal willingness to pay estimates for quality-of-life attributes using the conditional logit and random parameters logit model. In Table 3, the first column shows the results of the conditional logit model, while the second and third columns display results of the random parameters logit model.

The utility estimates obtained from the conditional logit model, as shown in Column 1 of Table 3, reveal that patients derive utility from all the attributes, preferring the most desirable levels. The least desirable level of each category is our base category, for instance, *no change in pain* for the pain category, etc. The direction of estimates represented by the sign of the coefficient is according to our expectations. A negative alternative-specific constant (ASC) indicates that patients are generally unhappy with their current situation, supporting our expectations. Our results further show that patients value improvements in pain, depression, mobility, self-care, and usual activities, with the highest satisfaction (utility) associated with improvements in usual activities, followed by mobility and self-care. Notably, the impact of improvements in depression has the least impact on utility. Anecdotal evidence suggests that patients often prioritize physical aspects over emotional well-being, which is reflected in our findings. The relatively small size of the depression coefficient emphasizes its minimal impact on utility compared to other attributes. The inverse relationship between utility and cost is confirmed by the negative sign on the cost coefficient, indicating that as the cost of the treatment option increases, the overall utility or satisfaction that patients derive from it decreases. This aligns with economic theory, which suggests that individuals tend to prefer options that offer greater benefits at lower costs. In practical terms, patients are less likely to choose an option if it comes with a higher financial burden. This is particularly relevant in the context of Nepal, where people have low incomes, and higher treatment costs can be a significant barrier for these patients.

Column 2 of Table 3 shows the results of the random parameters logit model for cancer patients with the associated standard deviations. Regardless of the signs, significant standard deviations indicate randomness of the parameter or taste variation among the patients. While signs of RPL estimates align with CL estimates, magnitudes increase, emphasizing the impact of attributes. Notably, heterogeneity is evident in certain attribute levels, such as alternative-specific constant, moderate pain, no depression, moderate problems in practicing self-care, and moderate problems in performing usual activities, represented by significant standard deviation. About 4 in 5 patients (80 percent) were unhappy with their current situation, as shown by the negative sign on the alternative-specific constant. However, the significant standard deviation in responses suggests that about 1 in 5 (20 percent) were satisfied with their current situation, potentially due to early-stage treatable cancer or any other reason. Most importantly, patients tended to agree on what they valued most: the best possible health outcomes, evident from the low variation in preferences for the highest attribute levels. Like CL estimates, the RPL estimates also show that patients gained the most satisfaction when they were able to carry out their usual daily activities on their own, followed by mobility, pain, and self-care activities. Similar patterns emerge in non-cancer patients (Column 3 of Table 3), favoring highly desirable outcomes. Even though many of them disliked their current situation, about 35 percent appeared content. Non-cancer patients valued improvements across all areas, but they gained the most from being pain-free and being able to carry out usual activities without problems.

We also performed analysis by the major types of cancer in Nepal: lung, breast, and cervical cancer. In the split analysis, cancer patients derived utility from fewer attributes; however, the preferences align with our expectations: higher preference was given to the best outcome, followed by the moderate and the least desirable outcome. Lung cancer patients, in particular, strongly valued pain relief and being able to perform their usual daily activities, besides disliking their current situation. For breast and cervical cancer patients, preferences about their current situation varied more widely. However, they gained the most satisfaction from being able to carry out daily tasks without problems, followed by being free of pain, having good mobility, and being able to take care of themselves.

Our analysis reveals no fundamental difference in utility estimates among different types of cancer patients. Except for lung cancer patients, the top priority is self-care and usual activities, while non-cancer patients prioritize pain reduction. Cancer patients suffer from extreme to moderate pain which hinders their day-to-day work. In our sample, the majority of the cancer patients are females; they have to perform daily household chores, and they also work in agricultural fields. Cancer pain hampers their progress; they have difficulty performing their work, which is why their highest utility comes from performing daily activities and reducing pain, and our statistics corroborate the fact. During pre-testing, we also engaged in “cheap talk” with cancer patients who were under treatment either as an outpatient or admitted in the hospital. We experienced that patients mostly focus on pain and self-care activities. A few of the outpatient females said, “Please give us something to reduce pain, so that we can work at home and in the fields.” Outpatients generally do not have many problems with mobility; therefore, they did not talk about that. However, inpatients, who are totally confined to their beds, emphasized mobility along with pain and usual activities.

### 3.3. Marginal Willingness to Pay

As described above, the discrete choice experiment method allows us to calculate marginal willingness to pay (MWTP) for different QoL attributes. The MWTP for improving quality of life is the amount of money an individual is willing to pay to attain an improvement in one of the attributes. Table 4 presents MWTP estimates for different QoL attributes for cancer and non-cancer patients.

Intuitively, we expect that patients would pay more for the most desirable outcome of each alternative. For instance, in the case of pain, the patients would be willing to pay more for *no pain* than for *moderate pain*. Except for self-care with lung cancer patients, all the MWTP estimates of Table 4 correspond to our hypothesis. Cancer patients exhibit the highest willingness to pay for the level of self-care activity attribute termed *no problems in performing self-care activities*. This is followed by *no pain* and *no problems in mobility*. On the contrary, non-cancer patients are willing to pay highly for *no pain,* followed by *no problems in performing self-care activities*. Due to the fear and suffering associated with cancer, we expect that cancer patients are willing to pay more than non-cancer patients. Except for *moderate depression* and *moderate problems in practicing self-care*, the estimates show that cancer patients are willing to pay at least NRS 50,000 more than non-cancer patients, depending on the attribute. Overall, cancer patients are willing to pay about NRS 2,610,853 for the alternative that contains the following attribute levels: *no pain, no depression, no problems in mobility, no problems in practicing self-care, and no problems in performing usual activities*. This combination of attributes presents the best alternative, which improves their quality of life to the level of a healthy individual. We can also interpret this amount [NRS 2,610,853] as the maximum amount that cancer patients are willing to pay for QoL improvement. We also estimated an overall willingness to pay for the best alternative among non-cancer patients, and they were willing to pay about NRS 2,054,838 for this alternative.

### 3.4. Sensitivity Analysis

In stated preference methods, respondents’ uncertainty about their choices can introduce bias if not properly addressed [27,28]. We use two data recoding approaches to handle choice uncertainty: eliminating uncertain choices and asymmetrically recoding them as the status quo alternative. In our case, after every choice set, we asked respondents, “How certain are you of your choice?” and the respondents were required to answer on a five-point Likert scale ranging from “very certain” to “very uncertain.” While performing the recoding, we combined “very certain” and “somewhat certain” answers to be one category showing certain choices of individuals, and “neither certain nor uncertain,” “somewhat certain,” and “very uncertain” to be one category representing uncertain choices.

Despite the different methods, the RPL estimates for quality-of-life attributes remain consistent with previous findings. The utility estimates exhibit the same trend as before, indicating that uncertainty does not significantly impact the results. Incorporating uncertainty does not substantially alter the willingness to pay estimates, reinforcing the robustness of our findings.

## 4. Discussion

The findings of this study align with the broader literature on health economics and patient preferences, particularly concerning the trade-offs between different QoL attributes. In this study, both cancer and non-cancer patients derive utility from improvements in key QoL attributes, but their preferences show marked differences, especially in terms of prioritization. Cancer patients, in particular, show a higher willingness to pay for improvements in self-care and usual activities, which can be linked to the debilitating nature of their condition that significantly impairs their daily functioning. This outcome is consistent with findings from studies on chronic illness patients, where the desire to regain autonomy and reduce physical limitations dominates decision-making processes [29].

The high utility derived from improvements in pain, mobility, and self-care reflects a well-established trend in the QoL literature. Physical suffering, particularly pain, is frequently prioritized over emotional or mental health improvements. The minimal utility cancer patients derive from addressing depression corroborates existing research suggesting that physical health often takes precedence over mental health concerns in clinical populations [30]. This is further supported by anecdotal evidence from the “cheap talk” pre-testing, where cancer patients, particularly those working in physically demanding roles such as agriculture, emphasized pain relief as critical to their ability to resume work.

The heterogeneity observed in the random parameters logit (RPL) model suggests significant variability in patient preferences, which mirrors findings in studies that emphasize the need for personalized treatment plans [31]. This study’s results reveal that while most cancer patients strongly prefer avoiding the status quo, a smaller subset (20% of cancer patients and 35% of non-cancer patients) show contentment with their current health state. This might be attributed to the fact that cancer patients in earlier, more treatable stages, or those who have adapted to their condition, may prioritize maintaining their current level of functioning rather than seeking improvement. Previous studies on health adaptation similarly suggest that patients in less severe stages of illness may be more accepting of their conditions [32].

Marginal willingness to pay (MWTP) estimates indicate that cancer patients are willing to pay significantly more than non-cancer patients for improvements in their QoL, particularly in reducing pain and enabling usual activities. This finding supports prior literature that shows cancer patients’ willingness to make substantial financial sacrifices for interventions that could restore normal functioning and relieve physical suffering [33]. Interestingly, the fact that cancer patients prioritize usual activities and self-care over pain in their willingness to pay may reflect their urgent need to regain autonomy and resume daily responsibilities, especially given the demographic characteristics of the sample, which includes many women working in agriculture.

## 5. Limitation

This study offers valuable insights but has some limitations. First, relying on hospital-based data and the lack of a population-based cancer registry in Nepal may affect sample representativeness. While the study covers a range of cancer patients, it may not fully reflect the preferences of those in remote or underserved areas with limited healthcare access.

Second, using a discrete choice experiment (DCE) to measure willingness to pay (WTP) involved hypothetical scenarios that may not mirror real-world decisions. Though DCEs are widely used in health economics, participants’ stated preferences may differ from their real behavior.

Lastly, the study focuses on five QoL attributes based on the EuroQol framework. While important, other factors like social support, access to care, and financial burden might also shape patient well-being. Expanding QoL measures could offer a more complete view of patient needs and preferences.

## 6. Conclusions

The primary objective of this study was twofold: first, ascertaining cancer patients’ preferences associated with different attributes of quality of life, and second, estimating their willingness to pay for improved quality of life. Using an individual-level dataset from Nepal and employing a random utility model, we found that cancer patients prefer the usual activities attribute the most. The second attribute that they value highly after usual activities is mobility. The rest of the attributes come afterwards. These utility estimates can be translated into willingness-to-pay values. Overall, cancer patients are willing to pay about NRS 2.6 million [about USD 26,000] for improving their quality of life. The individual estimates show that they are willing to pay highly for *no problems in performing usual activities,* followed by *no pain*. This shows that, among the quality-of-life attributes, daily activities and pain are the principal priorities for cancer patients.

Further analyses show that unlike cancer patients, non-cancer patients’ foremost priority is reducing pain, and they are willing to pay about NRS 1.8 million [about USD 18,000] for bringing their quality of life to par with the QoL of a healthy person. The presence of uncertainty can affect the results and perhaps create bias [27]; however, this situation did not arise in our case. We used two different methods to incorporate uncertainty in the data, and the results remained stable.

## Figures and Tables

**Table 1:** Attributes and levels.

Attributes	Levels
Pain	No pain; Moderate pain; No change in pain
Depression	No depression; Moderate depression; No change in depression
Mobility	No problems in mobility; Moderate problems in mobility; Confined to bed
Self-care	No problems in self-care; Moderate problems in self-care; Unable to practice self-care
Usual activities	No problems in performing usual activities; Moderate problems in performing usual activities; Unable to perform usual activities
Cost	0; 1000; 25,000; 50,000; 100,000; 175,000; 300,000; 500,000; 900,000; 1,200,000; 1,700,000; 2,500,000; 3,500,000

Data source: Nepal Study Center, UNM.

**Table 2:** Descriptive statistics of cancer and non-cancer patients.

Variable	Average	Cancer Patients	Non-Cancer Patients
Age (in years)	52.0	52.0	53.0
Patients		70%	30%
Inpatients (=1)	16.0%	15.0%	20.0%
Gender (Female = 1)	58.0%	61.0%	52.0%
Married	80.0%	82.0%	76.0%
Education			
No Schooling (=1)	53.0%	56.0%	46.0%
Bachelor’s (=1)	2.0%	1.0%	4.5%
*Ethnicity*			
Brahmin/Chhetri/Janajati	75.0%	69.0%	89.0%
Income			
<NRS 10,000	20.0%	24.0%	12.0%
NRS 10,001 to NRS 30,000	55.0%	55.0%	54.0%
>NRS 50,000	10.0%	7.0%	15.0%

Data source: Nepal Study Center, UNM.

**Table 3:** Utility estimates of QoL attributes.

Variable	CL (1)	RPL			
		Cancer (2)		Non-Cancer (3)	
	Choice	Mean	SD	Mean	SD
ASC	−1.21 ***	−2.50 ***	2.96 ***	−1.59 ***	4.13 ***
	(0.10)	(0.23)	(0.27)	(0.34)	(0.54)
No change in pain	ref.	ref.	ref.	ref.	ref.
Moderate pain	0.55 ***	1.08 ***	0.62 *	1.72 ***	0.17
	(0.08)	(0.15)	(0.35)	(0.31)	(0.15)
No pain	0.92 ***	1.56 ***	0.00	2.73 ***	−0.59
	(0.09)	(0.15)	(0.04)	(0.39)	(0.66)
No change in depression	ref.	ref.	ref.	ref.	ref.
Moderate depression	0.19 **	0.30 **	0.29	0.67 ***	0.37
	(0.08)	(0.13)	(0.39)	(0.26)	(0.38)
No depression	0.60 ***	0.83 ***	−1.07 ***	1.20 ***	1.31 ***
	(0.08)	(0.13)	(0.24)	(0.28)	(0.37)
Confined to bed	ref.	ref.	ref.	ref.	ref.
Moderate problems in mobility	0.43 ***	0.79 ***	−0.01	0.96 ***	1.67 ***
	(0.09)	(0.13)	(0.09)	(0.30)	(0.41)
No problems in mobility	0.70 ***	1.30 ***	−0.05	1.34 ***	−0.10
	(0.09)	(0.16)	(0.03)	(0.32)	(0.29)
Unable to practice self-care	ref.	ref.	ref.	ref.	ref.
Moderate problems in practicing self-care	0.52 ***	0.75 ***	−0.58 **	1.51 ***	0.19
	(0.09)	(0.15)	(0.28)	(0.34)	(0.12)
No problems in practicing self-care	0.73 ***	0.95 ***	−0.05	1.56 ***	0.16
	(0.08)	(0.13)	(0.07)	(0.33)	(0.17)
Unable to perform usual activities	ref.	ref.	ref.	ref.	ref.
Moderate problems in performing usual activities	0.59 ***	1.29 ***	−1.10 ***	1.67 ***	−0.22
	(0.07)	(0.16)	(0.33)	(0.29)	(0.26)
No problems in performing usual activities	0.92 ***	1.72 ***	–0.54	2.73 ***	2.41 ***
	(0.09)	(0.18)	(0.42)	(0.49)	(0.57)
Cost	−0.15 ***	−0.24 ***		−0.46 ***	
	(0.02)	(0.02)		(0.08)	
N	2730	2730		1173	

Data source: Nepal Study Center, UNM. Notes: *, **, *** denote statistical significance at 0.10, 0.05, and 0.01 level, respectively. N represents the number of observations. Clustered standard errors are in parentheses.

**Table 4:** MWTP estimates of QoL attributes.

Attribute	Cancer	Non-Cancer
Moderate pain	445,105 (315,083–575,127)	369,246 (221,942–516,549)
No pain	639,670 (500,291–779,048)	587,631 (405,651–769,610)
Depression		
Moderate depression	121,540 (18,954–224,126)	144,660 (29,409–259,910)
No depression	342,072 (232,074–452,070)	257,745 (120,485–395,005)
Mobility		
Moderate problem	323,782 (209,111–438,453)	207,256 (58,227–356,285)
No problem	532,864 (390,298–675,430)	287,531 (125,227–449,834)
Self-care		
Moderate problem	307,240 (187,138–427,341)	326,065 (180,603–471,528)
No problem	389,159 (279,284–499,035)	334,831 (189,941–479,721)
Usual activities		
Moderate problem	528,620 (393,334–663,907)	358,767 (215,243–502,291)
No problem	707,086 (560,442–853,730)	587,100 (375,214–798,986)

Data source: Nepal Study Center, UNM. Notes: Confidence Intervals in parentheses.

## Data Availability

Nepal Study Center (NSC), University of New Mexico, is the custodian of the data used in this study. Data will be made available upon formal request.
